# Design of Microscale Magnetic Tumbling Robots for Locomotion in Multiple Environments and Complex Terrains

**DOI:** 10.3390/mi9020068

**Published:** 2018-02-03

**Authors:** Chenghao Bi, Maria Guix, Benjamin V. Johnson, Wuming Jing, David J. Cappelleri

**Affiliations:** 1 School of Mechanical Engineering, Purdue University, West Lafayette, IN 47907-2088, USA; bi10@purdue.edu (C.B.); mguixnog@purdue.edu (M.G.); john1360@purdue.edu (B.V.J.); 2 A. Leon Linton Department of Mechanical Engineering, Lawrence Technological University, Southfield, MI 48075-1058, USA; wjing@ltu.edu

**Keywords:** mobile microrobot, magnetic actuation, tumbling locomotion

## Abstract

This paper presents several variations of a microscale magnetic tumbling (μTUM) robot capable of traversing complex terrains in dry and wet environments. The robot is fabricated by photolithography techniques and consists of a polymeric body with two sections with embedded magnetic particles aligned at the ends and a middle nonmagnetic bridge section. The robot’s footprint dimensions are 400 μm × 800 μm. Different end geometries are used to test the optimal conditions for low adhesion and increased dynamic response to an actuating external rotating magnetic field. When subjected to a magnetic field as low as 7 mT in dry conditions, this magnetic microrobot is able to operate with a tumbling locomotion mode and translate with speeds of over 60 body lengths/s (48 mm/s) in dry environments and up to 17 body lengths/s (13.6 mm/s) in wet environments. Two different tumbling modes were observed and depend on the alignment of the magnetic particles. A technique was devised to measure the magnetic particle alignment angle relative to the robot’s geometry. Rotational frequency limits were observed experimentally, becoming more prohibitive as environment viscosity increases. The μTUM’s performance was studied when traversing inclined planes (up to 60°), showing promising climbing capabilities in both dry and wet conditions. Maximum open loop straight-line trajectory errors of less than 4% and 2% of the traversal distance in the vertical and horizontal directions, respectively, for the μTUM were observed. Full directional control of μTUM was demonstrated through the traversal of a P-shaped trajectory. Additionally, successful locomotion of the optimized μTUM design over complex terrains was also achieved. By implementing machine vision control and/or embedding of payloads in the middle section of the robot, it is possible in the future to upgrade the current design with computer-optimized mobility through multiple environments and the ability to perform drug delivery tasks for biomedical applications.

## 1. Introduction

Microscale mobile microrobots have recently emerged as viable candidates for biomedical applications, taking advantage of their small size, manipulation and autonomous motion capabilities. Robotics at the micro- and nano-scale represent one of the new frontiers in intelligent automation systems. They face the challenges that imply working at low Reynolds numbers (viscous forces being dominant) [1] and the effective implementation of on-board actuation, sensing, power and conventional autonomous control techniques in an extremely small footprint [2]. Alternatives to on-board power and actuation are required at these size restrictions [3], demanding the use of advanced fabrication approaches such as microfabrication or 3D printing techniques, among others [4]. Targeted drug delivery is one of the key applications of these nano- and micro-robots [5].

As one moves down the length scale, the influence of adhesion and friction, which scales with surface area, becomes more pronounced, while inertia and applied forces are more unpredictable. To address these issues, several methods have been devised to actuate microrobots in dry conditions, including incorporating electrostatic principles [6] and thermal energy [7]. Crawling locomotion was achieved through induced bending deflection caused by electrostatic attraction and laser heating, respectively. For these actuation techniques, engineered surfaces or open environments are often required, limiting their practicality in biomedical applications. In such cases, an untethered microrobot that is adaptable to various environments and that does not need a specialized control substrate or open environment is the ideal solution. Magnetic actuation techniques have shown the most promise in achieving this solution.

Magnetically-actuated microrobots can be wirelessly controlled by external energy sources without the use of any special control surfaces [8,9,10]. Magnetic field gradients using customized magnetic coil systems [11,12,13] or an magnetic resonance imaging (MRI) scanner [14,15] have been commonly explored to actuate magnetic microrobots. These microrobots typically consist of magnetic bodies in fluidic environments to reduce the frictional forces experienced by the robots. A single neodymium-iron-boron permanent magnet was used as a mobile microrobot and was shown to translate with a rocking (stick-slip) motion by pulsing its magnetic field signals in [16,17,18,19]. For enhanced mobility in fluidic media, micro-scale spiral magnetic structures have been developed to cork-screw forward under a rotating magnetic field [10]. Both synthetic microrobots [20] and bio-hybrid microrobots [21] have been designed for rotating magnetic field actuation for drug delivery and fertility treatment applications, respectively. Rolling locomotion has also been used as an adaptive working mechanism for magnetic microrobots. A ball-shaped prototype, for example, has been fabricated to roll on surfaces [22,23], and a rolling magnetic microrobot named “RodBot” has been developed to manipulate objects in fluidic environments using non-contact methods [24,25]. The robots are all actuated with rotating magnetic fields. Recently, 3D printed magnetic microrobots inspired by ciliary microorganisms showed effective motion due to the non-axial symmetric beating motion of the cilia under stepping magnetic fields [26]. For dry environments, the “MagMite” and “PolyMite” designs are able to overcome the higher attractive forces and traverse dry surfaces by actuating two asymmetric magnetic micro-swing hammers with an oscillating magnetic field [27,28,29,30]. However, these robots require complex designs and microfabrication methods. Nanoscale swimming robots driven by oscillating and rotating magnetic fields have also recently been demonstrated [31,32]. A wide range of manipulation modes using magnetic mobile microrobots has been explored (e.g., contact-based pushing manipulation, noncontact fluidic manipulations, etc.) [33]. The potential to autonomously control them using vision-based control [34,35] has also been demonstrated. Team manipulation and swarm robotics is also being investigated to perform collaborative micromanipulation tasks as with mobile microrobots [36,37,38,39].

For many of the magnetic microrobots reviewed above, relative motion or constant contact exists between the agent and the substrate during the locomotion cycle. This behavior limits the mobility of these magnetic microrobots due to the increased resistance they experience. For microscale robots to operate successfully in real working environments, mobility is critical. This is because: (1) the acting magnetic force becomes dramatically weaker as the agent volume scales down; and (2) the resistance coming from a complex, biological fluid or surface can be very large when compared with the microrobot actuating power. Very high magnetic field strengths will be needed to overcome these resistances, which can be hard to achieve in practical applications.

An alternate form of microscale locomotion was explored by using a microscale tumbling magnetic robot with a dumb-bell structure with two oppositely-polarized magnetic bell parts in our previous work [40,41]. Its tumbling working mechanism, actuated with an alternating magnetic field, avoids the aforementioned relative motion and constant contact against the surface, achieving effective motion along non-smooth surfaces. Lower magnetic field strengths are needed to drive the robot due its tumbling locomotion mode. A second mode of operation (stick-slip) was also demonstrated for these robots for use in manipulation tasks. However, both stick-slip and sliding motion are difficult on non-planar geometries, such as bumps or trenches. Therefore, in this paper, we focus on a microrobot design, fabrication and actuation strategy to overcome these complex terrains in both wet and dry environments. We first introduce new microscale magnetic tumbling microrobots (μTUMs) fabricated by a versatile microfabrication approach that permits us to define different rotational axes and geometries of its magnetic ends for control with an external rotating magnetic field. The proposed μTUM is then quantitatively analyzed with theoretical methods, considering the unique resistance factors taking place at the microscale. μTUM prototypes with different particle alignment and motion performance under rotational magnetic fields, as well as various geometries at their magnetic ends, are tested to demonstrate the tumbling mechanism on the microscale in both wet (silicone oil, water) and dry environments. They show an increased dynamic response to an actuating external rotating magnetic field when compared to other rolling robots (i.e., RodBot). The optimal μTUM configuration is later tested for its ability to climb different inclined planes and traverse complex terrains. Finally, the paper is concluded with a discussion on present limitations and future work.

## 2. μTUM Robots

### 2.1. Design Overview

**Figure 1:**
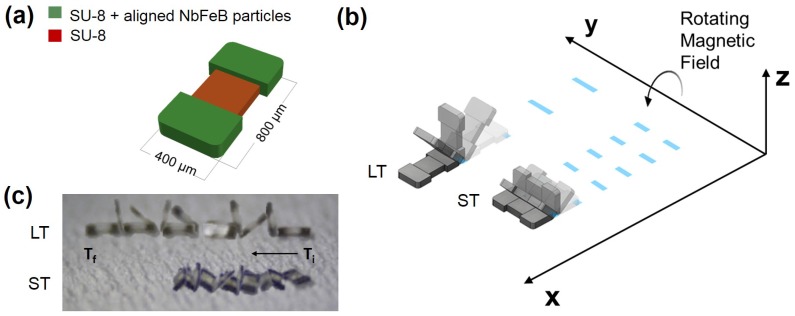
(**a**) Schematic of the microscale magnetic tumbling (μTUM) robot. The rectangular-shaped microrobot has embedded magnetic particles at each end. (**b**) Depending on the alignment of the magnetic particles, a rotating magnetic field will cause the μTUM to either lengthwise tumble (LT) or sideways tumble (ST). (**c**) Screen-shots from Supplemental Video S1 demonstrating both types of tumbling locomotion.

The magnetic microscale tumbling robot (μTUM) presented in this paper is rectangular-shaped with magnetic particles embedded in each of its two length-wise ends; see Figure 1. Permanent hard magnetic bodies are implemented instead of soft magnetic bodies, such as nickel, due to their generally higher magnetization values and their ability to be magnetically aligned in directions independent of their geometry. The robot’s magnetic features are aligned along the same direction and behave similarly under the presence of an external magnetic field. When the magnetic alignment of the field differs from that of the robot, a magnetic torque is induced on the robot until it is realigned with the field. Applying a continuously rotating magnetic field along a rotational axis parallel to the horizontal plane causes the robot to rotate about the same axis. If this rotating field is applied on a robot that is resting on a surface, the result is a forward tumbling motion, where the robot propels itself by continuously flipping end-over-end.

While a cylindrical form factor would result in a more predictable, precise rolling motion, such geometry would also reduce the robot’s responsiveness and performance. To travel at the same speed under a rotating magnetic field as a tumbling flat bar (rectangular) robot, assuming the robot operates with no slip, a rolling cylindrical robot needs to have a diameter that is equal to the bar robot’s length. In this case, the resultant cylindrical robot’s mass will be larger than that of the flat bar robot. The increased mass leads to more weight, making it harder for the robot to climb inclined surfaces. Additionally, curved three-dimensional geometry is difficult to fabricate at the microscale level using traditional techniques, and prior research has largely focused on flat form factors for hard-magnet micro-robots. For these reasons, a tumbling rectangular-shaped robot was chosen over a rolling robot to tackle rough terrain and unpredictable environments.

Flat, hard-magnet robots have been demonstrated in the past to operate in both dry and wet conditions [19]. Unlike the μTUM, however, these robots use rocking (stick-slip) motion under an alternating magnetic field, where contact between the robot and the surface is continually lost and regained with each oscillation. Though the continuously rotating field used for the μTUM is harder to implement than an alternating field, the trade-off is that the tumbling robot always has a point in contact with the ground, provided that there are no sharp drop-offs or cliffs in its path. This sustained contact means that the μTUM design can take advantage of the constant adhesion and frictional forces between itself and the surface below it to climb steep inclined terrains. At the microscale, where the effect of surface forces begins to surpass the effect of volumetric forces, the magnitude of these two forces can often exceed that of gravitational force.

**Figure 2:**
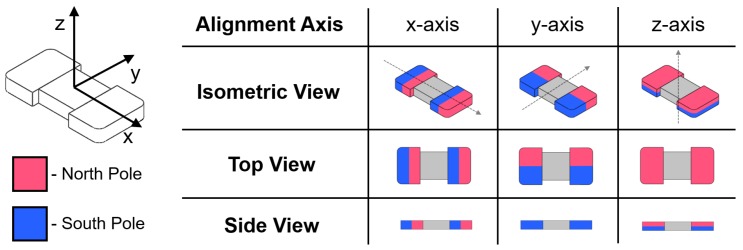
Visualization of internal magnetic alignment along the robot’s three major axes.

**Figure 3:**
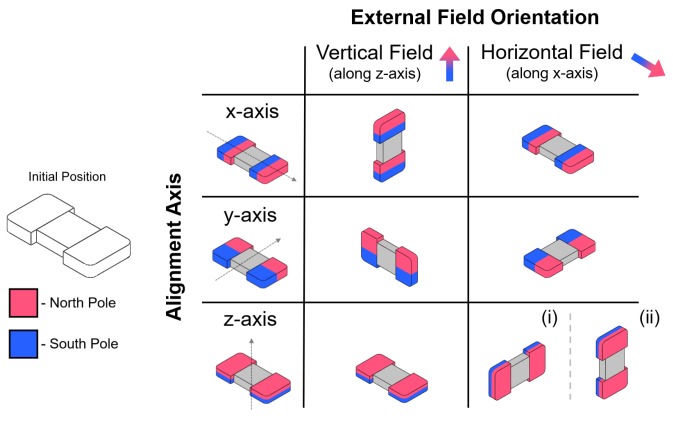
Equilibrium resting states of each major axis alignment configuration under horizontal and vertical external magnetic fields. Note: for z-axis alignment, both (i) and (ii) are possible under the same conditions, but (i) is in a lower energy state than (ii) and thus more likely to occur in practice.

Two distinct tumbling motions, a lengthwise tumbling (LT) motion and a sideways tumbling (ST) motion (Figure 1b) can be observed depending on the direction of the µTUM’s internal magnetic alignment. The robot’s hard magnet design allows its magnetic poles to be aligned along any direction, so long as the fabrication method permits it. When the poles are aligned along one of the robot’s major geometric axes, as defined in Figure 2, μTUM will either tumble about its length or tumble about its width. Under the same external rotating field, a lengthwise tumbling μTUM will travel faster than a sideways tumbling μTUM (see Supplementary Video S1). The former design, however, also requires more force to raise up from a flat initial resting position, due to its longer lever arm. Since speed was deemed to be more important for locomotive purposes, the lengthwise tumbling design was chosen to be the primary configuration of μTUM. To determine the correct magnetic alignment for enabling this type of tumbling motion, the equilibrium states of several μTUM alignment configurations were analyzed. The robot’s initial position is defined in Figure 3, and all forces apart from magnetic torque are ignored in this static analysis. It can be observed that there is only one configuration where the robot consistently moves to the upwards lengthwise position necessary for lengthwise tumbling. This case occurs when the robot is magnetically aligned along its x-axis and is influenced by a vertical horizontal field. There is another configuration in which the upwards lengthwise position is seen (Figure 3(ii)), but in this case, an upward sideways position (Figure 3(i)) is also possible under the same field orientation. Because the sideways position is at a lower energy state than the lengthwise position (due to the sideways position’s lower center of mass), this other configuration tends to result in sideways tumbling. Pure lengthwise tumbling is only guaranteed in the former case, where the robot is magnetically aligned along its geometric x-axis. As a result, the robots used for the majority of the experiments are magnetically aligned in this manner and are optimized for lengthwise tumbling.

Several other design variations of the geometries of the magnetic body sections were also developed to explore how this geometry might affect its tumbling performance. The number of corners, the sharpness of the corners and the protrusion of the corners were varied to see if the robot’s performance would be significantly affected by any of these factors. The effect of symmetry was also explored by fabricating robots with ends of slightly different lengths. For testing purposes, the rounded rectangle geometry was chosen as the default design, due to its uniform flat edges and predictable behavior, and the other designs were compared against it. For all the design configurations considered, the mid-section of the robot was kept non-magnetized in order to explore the future possibility of embedding a payload in this area of the robot.

### 2.2. Modeling

One of the first considerations for the tumbling robot is whether the driving external magnetic field is strong enough to rotate it up from an initial resting position, where forces due to gravity, electrostatic attraction and surface adhesion must be overcome. A static analysis of the forces on the robot was conducted to determine the minimum external field strengths required for upwards rotational movement. For modeling purposes, the various constituents of microscale surface adhesion, such as intermolecular bonding, van der Waals interactions and capillary forces, are lumped together into a single adhesive force $F_{a}$. The modeled robot’s magnetic alignment is assumed to be optimized for lengthwise tumbling, and the external magnetic field is assumed to be uniform and time-invariant.

**Figure 4:**
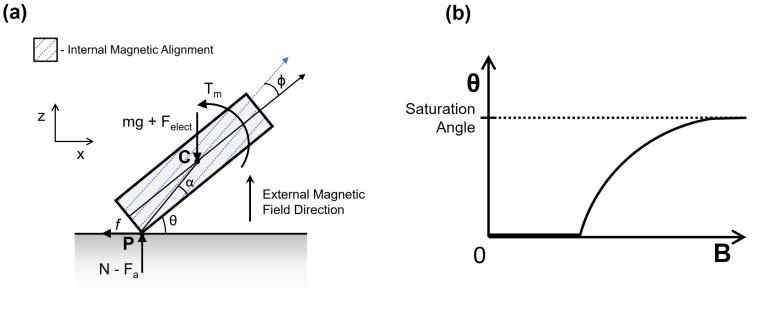
(**a**) Force diagram: Point C is the center of mass and Point P is the contact point. Note that adhesion force will act on the center of mass if the robot is resting flat on the ground. (**b**) General plot of magnetic field strength versus robot orientation angle.

When the internal magnetic alignment of the robot differs from that of the external field, a magnetic torque is induced on the robot. This torque can be described as:

(1) $${\vec{T}}_{m}=V_{m}\vec{M}\times \vec{B}$$

where $V_{m}$ is the magnetic volume of the robot, $\vec{M}$ is the magnetization of the robot and $\vec{B}$ is the magnetic field strength, or flux density, of the external field. Though additional forces may act on the robot due to magnetic field gradients, such forces are not considered in the model because no significant field gradients were applied during experimentation. From Equation (1), it can be seen that the maximum magnetic torque will occur when the robot and the external field alignments are perpendicular to each other. Assuming that the robot is initially resting on a flat horizontal surface, this relationship indicates that the external field should be vertically aligned to obtain the maximum possible magnetic torque on the robot. Additional torque due to gravitational, electrostatic and adhesive forces counteract this applied magnetic torque and hinder the robot from rotating upwards. When the magnetic torque is kept static, all the torques balance out, and the robot comes to rest at an equilibrium angle. One end of the robot will maintain contact with the surface while the other end rotates upward. The contacting end of the robot can be considered as a no-slip point that is pinned to the surface. The resultant side-view free-body diagram can be seen in Figure 4a.

In practice, it is observed that the actual magnetic alignment of μTUM does not match the desired magnetic alignment exactly, due to the alignment errors in the fabrication method used. An alignment offset angle ϕ, defined as the angular difference between the robot’s actual alignment direction and the desired alignment direction, is introduced in the model to account for this discrepancy. Due to varying alignment offset angles, it is possible for different μTUM robots to stabilize at different equilibrium orientations, despite having identical geometries, applied fields, material properties and operating environments. Determining the alignment offset angle ϕ is critical because the robot will eventually transition from the desired lengthwise tumbling motion to an undesired sideways tumbling motion as ϕ increases from 0${}^{\circ }$–90${}^{\circ }$.

Evaluating a moment equilibrium about the pinned contact point P in Figure 4a yields the following equations:

(2) $$\Sigma M_{P}=0=T_{m}-\left(mg+F_{elect}\right)\frac{L}{2}\cos\left(\theta +\alpha \right)$$

(3) $$V_{m}|M||B|\cos\left(\theta +\phi \right)=\left(mg+F_{elect}\right)\frac{L}{2}\cos\left(\theta +\alpha \right)$$

(4) $$\left|B\right|=\frac{\left(mg+F_{elect}\right)\frac{L}{2}\cos\left(\theta +\alpha \right)}{V_{m}\left|M\right|\cos\left(\theta +\phi \right)}$$

(5) $$|B_{min}|=\frac{\left(mg+F_{elect}+F_{a}\right)\frac{L}{2}\cos\alpha }{V_{m}\left|M\right|\cos\phi }$$

where *L* is the length of the robot, θ is the equilibrium orientation angle of the robot, ϕ is the magnetic alignment offset angle and α is the angle between the corner of the robot and its center of mass. $F_{a}$ and $F_{elect}$ are the adhesive and electrostatic forces respectively between the robot and the surface on which it rests. Equation (4) is valid when the orientation angle θ of the robot is greater than $0^{\circ }$, and Equation (5) is valid when the robot is flat against the surface and is just beginning to rise. The primary difference between the two equations is that the latter equation contains an adhesion force term while the former equation does not. This change is necessary because adhesion acts on the robot’s center of mass instead of the pinned point *P* when the robot is resting flat on the surface. Together, the two equations describe the relationship between the strength of the external magnetic field and the robot’s resultant orientation, with Equation (5) approximating the minimum field strength required to raise the robot up from the surface. Since the model only considers static equilibrium positions, the effects of dynamic forces such as viscous drag are neglected.

A simple measurement of when μTUM begins to slip on a surface of interest can be used to determine the unknown electrostatic and adhesion forces in Equations (2)–(5). The robot is laid flat on the horizontal surface, and the surface is then inclined until the robot begins sliding. At this point of slippage, the force of the robot’s weight down the incline matches the force of static friction, and the sum of the electrostatic and adhesive forces can be written as:

(6) $$F_{a}+F_{elect}=\frac{mg\sin\gamma }{\mu_{s}}-mg\cos\gamma$$

where angle γ is the surface inclination angle at which μTUM is observed to slip.

To increase the usefulness of Equation (6), several assumptions can be made about the nature of the electrostatic and adhesive forces. In dry environments, electrostatic force is significant for non-conductive objects because there is no fluid to dissipate individual charges. The opposite is true for fluid environments, where the presence of fluid makes the electrostatic force much smaller. On rough surfaces, adhesive force is negligible because high surface roughness reduces the contact area between the robot and the surface. The opposite is true for smooth surfaces, where adhesive force is significant due to large contact areas. Therefore, the left side of Equation (6) can be reduced to just the electrostatic force $F_{elect}$ in dry, rough environments, and it can be reduced to just the adhesive force $F_{a}$ in wet, smooth environments. An additional assumption is that the electrostatic force remains constant regardless of the robot’s orientation. Though it is known that this force is proportional to the distance between two objects, the change in distance between the surface and the robot as it rises is relatively small in comparison to the electrostatic force’s overall area of effect. The result of these assumptions is utilized in Section 4.1 to predict the robot’s orientation under varying external field strengths.

To determine the unknown alignment offset angle ϕ in Equations (4) and (5), the maximum equilibrium angle θ that is observed experimentally can be used. As the external field strength increases, the robot asymptotically approaches a saturated maximum equilibrium angle. This behavior is illustrated schematically in Figure 4b. The right side of Equation (3) becomes nearly constant at higher field strengths, with the equilibrium angle θ approaching the constant saturation angle. In order for the left side of Equation (3) to also remain constant as the field strength increases, the cosine term must approach zero, and the following relationship can be derived:

(7) $$\theta_{max}=90^{\circ }-\phi$$

This relationship indicates that the offset angle ϕ can be determined by measuring the robot’s maximum equilibrium orientation angle $\theta_{max}$. Such a measurement can be performed by placing μTUM slightly above the center of a strong magnet, ideally where the magnet’s field is vertical, and measuring the robot’s resultant orientation angle θ. This simple process allows the alignment offset angle ϕ to be approximated without the need for complex measuring equipment. The angle can then be used to predict μTUM performance using Equations (4) and (5) and evaluate the success of different fabrication methods, where a smaller offset angle indicates less alignment error.

## 3. Fabrication

**Figure 5:**
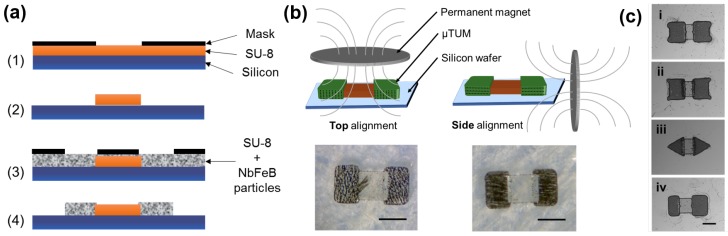
Fabrication process of microscale magnetic tumbling motors (μTUM). (**a**) Photolithography process followed for the the fabrication of μTUM. (**b**) Magnetic particle alignment techniques. Magnets located above the robot result in alignment along the geometric z-axis and sideways tumbling (ST); magnets located beside the robot result in alignment along the geometric x-axis and lengthwise tumbling (LT). (**c**) Optical images of the different geometric variations on the μTUM ending sections that have been explored: (i) rounded corners, (ii) sharp corners, (iii) triangles and (iv) (asymmetric) rounded rectangles. Scale bar, 300 μm.

The μTUM’s body is a polymeric-based microstructure consisting of (i) two sections with embedded neodymium-iron-boron (NbFeB) particles at the ends and (ii) one non-magnetic bridge part connecting them (Figure 5a). In order to obtain a better response to the magnetic fields, the NbFeB particles are conveniently aligned during the fabrication process itself (Figure 5b). μTUM fabrications are based on a simple and versatile two-step photolithography process, where the non-magnetic middle section is first photo-patterned by using bare SU-8 50 (Microchem Inc., Westborough, MA, USA) (Figure 5a, Steps 1 and 2), and the μTUM ends are then fabricated by SU-8 doped with NbFeB particles (Magnequench MQFP 5 μm, Neo Magnequench, Toronto, Canada) with a concentration of 10 gr/50 mL (Figure 5a, Steps 3 and 4). To pattern the middle polymeric section, the standard recommended protocol for structures of 100 μm in thickness was followed (www.microchem.com). The SU-8 50 is spin-coated at 1000 rpm for 30 s and then undergoes a soft baking process consisting of two consecutive steps of 10 min at 65 ${}^{\circ }$C and 30 min at 95 ${}^{\circ }$C in order to evaporate the solvent. The full wafer is then exposed in a mask aligner (UV (350–400 nm), Suss MA 6 Mask Aligner, SUSS MicroTec AG, Garching near Munich, Germany) by using a mask with a design corresponding to the middle section of the robot. A post-exposure bake (1 min at 65 ${}^{\circ }$C and 10 min at 95 ${}^{\circ }$C) is performed before the removal of the non-polymerized SU-8 with SU-8 developer (Microchem Inc.).

For the magnetic sections, the standard protocol was adjusted by including a pre-alignment step during the soft curing process (Figure 5b). By placing two permanent magnetic discs (3 inches in dia. × 1/8 inch-thick NdFeB, Grade N42, K & J Magnetics Inc., Plumsteadville, PA, USA) vertically next to the wafer (Figure 5b), a μTUM alignment along its geometric x-axis was obtained, resulting in a lengthwise tumbling (LT) motion (Figure 5b, right). However, when the same two magnets are located above the silicon wafer during soft curing, the resultant robots yield a sideways tumbling (ST) motion (Figure 5b, left). The type of tumbling motion that is observed in the finished robot is determined by the position of the two magnetic discs during the fabrication process.

Due to the flexibility of the fabrication method presented, where hard magnets can be formed out of any two-dimensional shape, several different geometries for μTUM’s magnetic ends were explored. Four types of geometries were considered in total: (i) rounded corners, (ii) sharp corners, (iii) triangles and (iv) rounded rectangles (Figure 5c). For the rounded rectangles geometry, two different cases were considered: a symmetric version (Figure 5b, right) and an asymmetric version (Figure 5c(iv)).

## 4. Experimental Results

**Figure 6:**
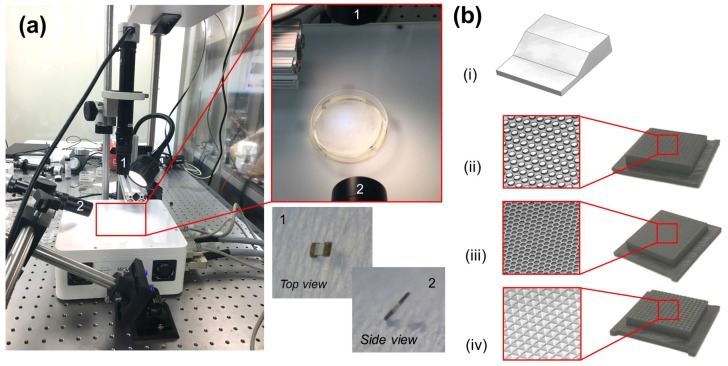
(**a**) The MFG-100 system with the top camera (1) and side camera (2). The paper surface inside a Petri dish at the center of the workspace. Top and side views of a μTUM as seen through the cameras. (**b**) Qualitative diagram of the various terrain geometries designed and fabricated: (i) inclined plane, (ii) cylindrical bumps, (iii) honeycomb and (iv) knurled.

The driving external magnetic fields are generated by the MFG-100 system (MagnebotiX AG, Zurich, Switzerland, magnebotix.com) as shown in Figure 6a. This system is capable of generating up to a 40 mT field strength and field gradients of up to 2 T/m at the center of the workspace, which is about 10 mm in diameter. The power unit of the field generator can supply up to 20 A currents and uses eight coils at a time to generate the specified field or gradient in the workspace. Rotational fields of frequencies up to 2000 Hz at 2 mT and 100 Hz at 8 mT can also be generated in the workspace. The control software communicates with the electronics hardware to specify the field strength, gradient and frequency of rotational fields in the workspace. This software can also control the yaw, roll and pitch of the field’s rotational axis, which allows for the steering control of μTUMs. Real-time imaging is captured by an overhead camera and a side camera aligned at 60° with the out of plane axis. Complementary metal oxide semiconductor (CMOS) cameras (Basler puA1600-60uc, Basler AG, Ahrensburg, Germany, baslerweb.com) along with microscope lenses (Edmund VZM 450i, Edmund Optics, Barrington, NJ, USA www.edmundoptics.com) of adjustable magnification are used in both the axes to record the motion of the robots.

The default design of the μTUM robot (rounded rectangle geometry with alignment tuned for lengthwise tumbling) was tested on a dry paper surface (Kimwipes, Kimtech Science, Kimberly-Clark Corporation, Irving, TX, USA) in order to evaluate its performance under varying field rotation frequencies and surface inclination angles. The external field strength was kept constant at 10 mT for all dynamic experiments. A paper surface within a dry environment was used for the majority of the characterization tests due to its challenging nature, where beneficial damping and buoyancy forces are minimal and friction forces are high. Additional performance tests were conducted in both water and silicone oil to gauge and characterize robot capabilities in fluid environments of varying viscosity.

Much like the geometric variants of μTUM, several terrain geometries were also developed to see if the presence of curved surfaces, small inclined surfaces and holes in the terrain would significantly affect the robot’s performance (Figure 6b). These terrain geometries include a pattern with cylindrical bumps, a honeycomb pattern and a knurled pattern. They were fabricated using a Form 1+ SLA 3D printer from Form Labs (www.formlabs.com).

### 4.1. Static Analysis Tests

To test the validity of the static model discussed in Section 2.2, the μTUM’s orientation under varying external field strengths was predicted and then juxtaposed alongside experimental data. For a rough paper surface within a dry environment, the parameters listed in Table 1 apply. Due to the reasoning detailed in Section 2.2, electrostatic force is assumed to be constant and adhesive force is assumed to be negligible. The saturated equilibrium angle $\theta_{max}$ was measured to be 63${}^{\circ }$, indicating that the magnetic alignment offset angle ϕ is 27${}^{\circ }$. Additionally, the robot was observed to begin slipping on the paper at a surface inclination angle of 70${}^{\circ }$, indicating an electrostatic force of $F_{elect}\approx 5.48\times 10^{-6}$ N.

**Figure 7:**
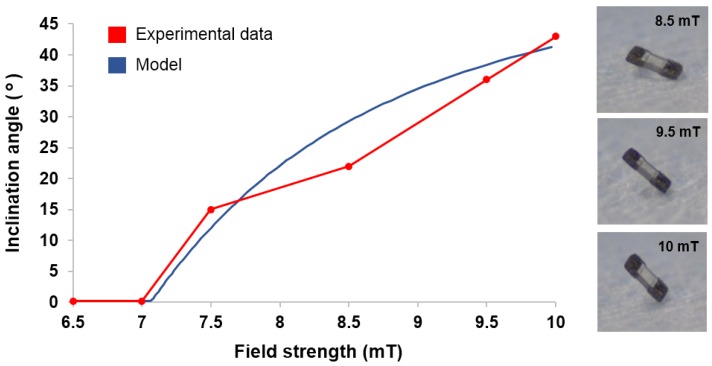
Model and experimental equilibrium orientation angle θ at varying field strengths (Results also shown in Supplemental Video S1).

Under a static vertical magnetic field, the robot’s equilibrium orientation angle θ was recorded for field strengths varying from 0–10 mT (Figure 7). The experimental angles closely matched those that were predicted by the model, and a similar minimum field strength of roughly 7 mT was required to raise the robot from its initial position.

### 4.2. Tumbling Locomotion Tests

**Figure 8:**
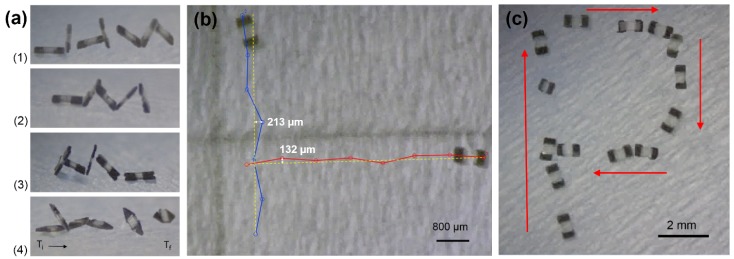
μTUM locomotion tests: flat paper substrate, dry environment, with 10 mT field strength at 0.5 Hz. (**a**) Images of trials for μTUM’s with different end variations: (1) rounded rectangle, (2) asymmetric rounded rectangle, (3) rounded corners and (4) triangle shape. (**b**) Trajectories (blue/red) of μTUM with respect to an ideal 5 mm-long straight line trajectory (yellow); the maximum trajectory drift for each is reported. (**c**) Default rounded rectangle μTUM design traversing the P-shaped trajectory. These results can be viewed in Supplemental Video S2.

The different geometric variants of the μTUMs were tested for their tumbling locomotion capabilities along the same paper surface in dry environmental conditions (Figure 8a). No significant differences were observed for robots with the rounded corners design and robots with the asymmetric variant of the default rounded rectangle design. However, the rounded corners robots were observed to be slightly faster than the default robots due to their longer length. Robots with the sharp corners design were also observed to be slightly faster than the default robots, but their corners had a tendency to hook into the fibers of the paper substrate. While this hooking action may potentially be beneficial for climbing steep inclines, it is often detrimental and causes the robots to remain anchored to a particular spot. Robots with the triangle design were observed to be vertically unstable and frequently tilted over to their side. This behavior likely occurred because their centers of mass were not perfectly aligned with their endpoints. The result is that robots with the triangle μTUM design cannot maintain a stable tumbling motion and move forward in an erratic manner.

To assess the open-loop trajectory accuracy of the μTUM, the default rounded rectangular version was commanded to translate along a 5 mm-long straight line path in both the vertical and horizontal directions. Figure 8b shows these open-loop trajectories. The maximum deviations from the straight line paths were 213 μm and 132 μm for the vertical and horizontal tests, respectively.

The μTUM with the default rounded rectangle design was also demonstrated to be steerable along a desired trajectory. Manually adjusting the pitch and the yaw of the external field’s rotational axis in increments of 90${}^{\circ }$ allowed the robot to travel in a roughly P-shaped trajectory (Figure 8c). The input was open-loop, and no closed-loop control was implemented.

**Figure 9:**
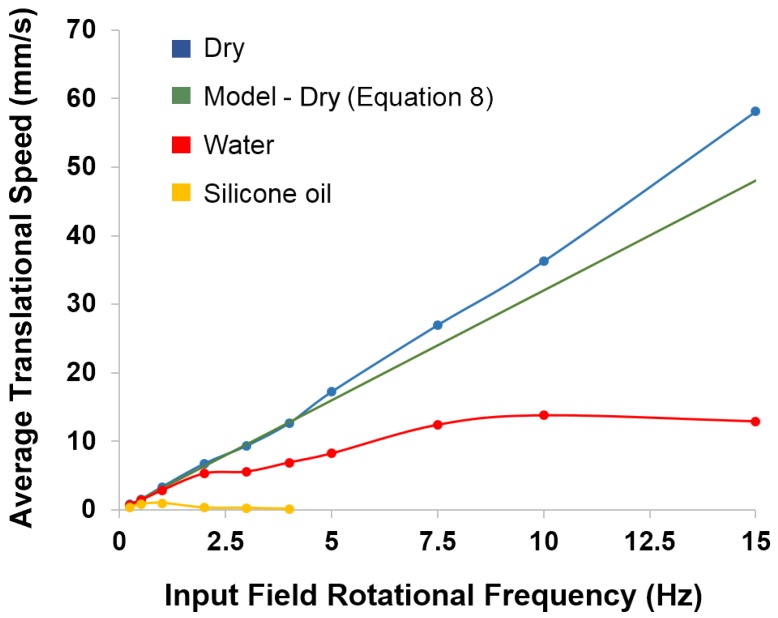
Experimental and modeled average translational speed under varying field rotational frequencies for various conditions. Results also shown in Supplemental Video S3.

Figure 9 plots the average translational speed of a default μTUM robot under varying field rotational frequencies for a variety of environmental conditions. It can be seen that the translational speed of the robot in dry air on paper increases roughly linearly as the rotational frequency of the external field increases. The robot was observed to be tumbling without slipping in this case. It follows that the robot’s average translational speed $\bar{v}$ should be approximately equal to four-times the body length (L) multiplied by the field rotational frequency $f_{rot}$:

(8) $${\bar{v}}_{no\;slip}=4Lf_{rot}$$

since the robot tumbles over its length twice when the magnetic field completes one full rotation. At higher field rotational frequencies, it can be seen that the experimental speeds are slightly higher than the predicted speeds. This discrepancy is likely because higher rotational frequencies can produce undesired magnetic field gradients in the MagnebotiX machine, which will apply a pulling force on the robot. A maximum translational speed of 58.1 mm/s was measured, but the upwards trend in Figure 9 suggests that the speed of μTUM will continue to increase as the external field’s rotational frequency increases above 15 Hz. On a substrate within a water environment, the presence of buoyancy forces leads to lower frictional forces, since friction is proportional to the total normal force. This reduced friction causes μTUM to slip on the substrate, and the resultant relationship between the robot’s translational speed and the field rotational frequency fails to follow a linear curve. At higher frequencies, the translational speed of the robot becomes noticeably saturated at 13.6 mm/s. On a substrate within a silicone oil environment, higher viscosity and larger drag forces lead to phase lag between the orientation of the robot and the orientation of the external magnetic field. As the external field’s rotational frequency increases, the larger drag forces prevent the μTUM from keeping up, and the robot begins oscillating in place instead of tumbling forward. This behavior occurs at field rotational frequencies of 2 Hz and above. Higher buoyancy forces in silicone oil result in even more slip, and the average translational speeds in this medium are much lower than in the others, with the maximum translational speed measured in silicone oil being 0.975 mm/s.

### 4.3. Inclined Plane Traversal Tests

**Figure 10:**
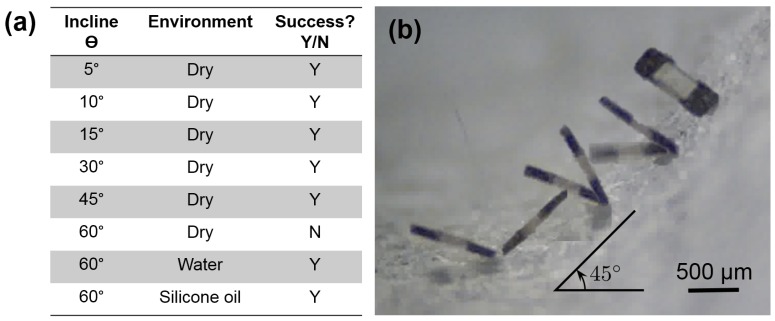
Inclined plane traversal tests. (**a**) Table of results for different inclination angles and environments; (**b**) images of the μTUM traversing a 45${}^{\circ }$ angle in dry conditions (10 mT field @ 0.5 Hz). Please see Supplemental Video S4 for video footage of these tests.

Tests to determine if the default design μTUM can traverse an incline at various angles were performed and evaluated on a pass/fail basis. The results are reported in Figure 10a. The dry air incline tests received more focus because the lack of beneficial buoyancy forces and the significant presence of electrostatic forces make the climb significantly harder. It was observed that the robot can go over a maximum inclination of 45${}^{\circ }$ in dry conditions on paper. Due to the additional buoyancy force and dissipation of electrostatic charges in the denser liquid mediums, the robot was shown to be capable of climbing inclines of at least 60${}^{\circ }$ in water and silicone oil. The possibility that the robot could be swimming was ruled out after it was observed that the robot did not move forward after losing contact with the surface (see Supplementary Video S3). Figure 10b shows images of the μTUM climbing up a 45${}^{\circ }$ angle in dry conditions. It does not slip on the terrain in dry conditions until it reaches an incline angle that it cannot traverse. When μTUM climbs inclines in either water or silicone oil, however, it tends to roll and slip along the incline, traveling at reduced overall speed.

### 4.4. Complex Terrain Tests

**Figure 11:**
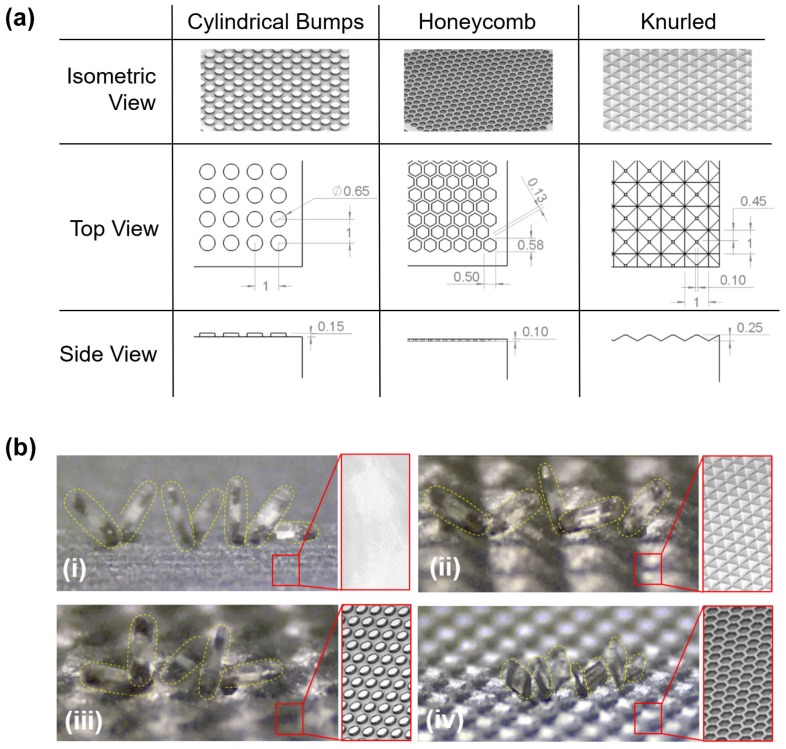
(**a**) Dimensions of the terrain geometries explored (all dimensions in millimeters); (**b**) μTUM traveling over different terrains in dry environments, with a 10 mT field strength @ 0.5 Hz. (i) Flat paper; (ii) cylindrical bumps; (ii) knurled surface; (iv) honeycomb terrain (side-ways tumbling mode). Please see Supplemental Video S5 for video footage of these tests.

Figure 11 presents the μTUM’s performance over various complex terrains. Tests were conducted on a flat paper surface and three 3D printed complex terrains, all in dry conditions. Figure 11a details the dimensions of each of the terrain features. Performance over the three complex terrains was similar, with mean velocities slightly slower than that of the flat surface. This can be attributed to the fact that the complex terrain surfaces bump or tilt the robot off to the side during the course of travel, as well as the difference in surface material.

Utilizing the default lengthwise tumbling mode, μTUM was able to travel over the knurled and cylindrical bump terrains seen in Figure 11b(ii,iii), respectively. However, it had difficulty getting up from its initial resting position on the honeycomb terrain. It is believed that concave pores, such as the honeycomb holes, increase the adhesive force between the robot and the surface when they are roughly the size of the robot’s cross-sectional area. After switching the default μTUM robot with a μTUM configured for sideways tumbling, the honeycomb terrain became much easier to traverse, as seen in Figure 11b(iv). With a smaller lever arm to rotate, the magnetic torque required to break the attractive surface forces decreased. The sideways tumbling μTUM variant, as a result, was able to traverse through honeycomb terrain under the same magnetic field strength and rotational frequency that the default design could not.

## 5. Discussion and Conclusions

The μTUM microrobot was able to exhibit tumbling movement through three different mediums under varying external field and surface conditions. As these environmental parameters were changed, several trends were observed. Increasing the drag coefficient of the surrounding environment limited the maximum field rotational frequency that the robot could follow. The dampened speeds observed in high-drag environments, however, also made the robot easier to observe and control. Increasing the density of the surrounding environment lowered the minimum field strength required to rotate the robot upwards from an initial resting position, due to larger buoyancy force. The reduced normal force between the robot and the surface, however, also reduced friction force and made the robot more prone to slipping. In contrast, increased attractive forces between the robot and substrate raise the minimum field strength required for initial upwards rotation and make the robot less prone to slipping. In dry environments, the drag and buoyancy forces are both negligible, and high tumbling speeds can be obtained. This advantage occurs at the cost of high external field strength requirements, due to the presence of electrostatic attraction between the robot and the substrate. The opposite behavior is observed in wet environments, where the drag and buoyancy forces are both significant and electrostatic charges are dissipated into the fluid. The various trends that occur as the μTUM traverses through different environments presents challenges in closed-loop control and optimal pathfinding that will need to be addressed in the future.

The experiments demonstrate that highly viscous fluids, such as silicone oil, impose limitations on the robot’s maximum rotational frequency, and low density mediums, such as air, impose limitations on the steepest incline that can be traversed. Minimizing slip, weight and the effect of electrostatic charges is crucial for reducing the effect of these limitations and optimizing traversal through multiple terrains. There is a need to minimize the slip experienced by the outer edges of the robot while simultaneously avoiding the hooking action observed in robots with sharp corners. There is also a need to reduce the adhesive force on the main body of the robot. Options for making such improvements include increasing the surface roughness of the μTUM, increasing the contact area of its outer edges and decreasing the contact area of the robot’s main body.

Of the four different types of robot geometries fabricated, the rounded corners and rounded rectangle designs were the most effective at maintaining consistent forward movement. Excessively sharp corners were found to increase the likelihood of the robot hooking into rough surfaces and getting stuck. Decreasing the number of corners reduced vertical stability in dry conditions and tumbling motion could not be achieved for triangular end geometries. From the three different complex terrains fabricated, it was observed that small concave surface features adversely affected the robot’s performance by increasing the adhesion force between the robot and the substrate.

Difficulties were encountered in measurements and modeling due to the anisotropic nature of adhesion and frictional forces on rough surfaces at the micro-scale. μTUM performance can vary significantly depending on the material properties of initial starting point. In the future, higher input field strengths can be used and magnetic particles with higher magnetization can be incorporated to reduce the overall effect of these forces. Low yield rates were also observed during fabrication due to the nature of the alignment magnets used. The field lines emanating from its poles are spread out and not necessarily oriented in the desired direction. A Helmholtz coil can be used instead of simple permanent magnets to generate nearly parallel alignment lines. In the meantime, the technique developed in Section 2.2 allows the alignment offset angle to be easily measured and used to determine the minimum field strength required for tumbling movement.

Future work will focus on dynamic modeling of the μTUM that can predict its motion trajectories over complex terrains, as well as addressing the unique challenges present at the interface of distinct environments. Capillary forces found in air-water interfaces, for example, can induce significant lateral forces on the robot that need to be considered [42]. Additional goals include developing vision-based closed-loop control for precisely navigating the robot to a desired destination and also revisiting the new μTUM design for micromanipulation tasks. Alternate designs for the mid-section of the robot, kept non-magnetized in this work, will be explored, as well. Replacing this area with a compliant material or a dissolvable payload could lead to improved dynamic behavior and in vivo drug delivery, respectively, with far-reaching potential in micro-object manipulation and biomedical applications.

## Figures and Tables

**Table 1:** Static inclination angle model parameters. The density of SU-8 (1190 g/m${}^{3}$) and NdFeB (7500 g/m${}^{3}$) were used to calculate mass.

Description	Value	Units
μTUM Length (*L*)	$0.8\times 10^{-3}$	m
Mass (*m*)	$1.6071\times 10^{-7}$	kg
Electrostatic Force ($F_{elect}$)	$5.4775\times 10^{-6}$	N
Static Friction Coefficient ($\mu_{s}$)	0.3	-
Geometric Offset Angle (α)	14.063	${}^{\circ }$
Magnetic Alignment Offset Angle (ϕ)	27	${}^{\circ }$
Magnetic Volume ($V_{m}$)	$2.9\times 10^{-11}$	m${}^{3}$
Magnetization (*M*)	15,000	A/m

## Supplementary Materials

The following are available online at www.mdpi.com/2072-666X/9/2/68/s6: Supplemental Video S1: μTUM lengthwise and sideways tumbling modes and inclination angle tests; Video S2: μTUM locomotion tests; Video S3: μTUM locomotion in different environmental conditions; Video S4: μTUM inclined plane traversal tests; and Video S5: μTUM traveling over different terrains.

Supplementary File 1

Supplementary File 2

Supplementary File 3

Supplementary File 4

Supplementary File 5

Supplementary File 6
